# Development of Endotoxin Tolerance Does Not Influence the Response to a Challenge with the Mucosal Live-Attenuated Influenza Vaccine in Humans *In Vivo*

**DOI:** 10.3389/fimmu.2017.01600

**Published:** 2017-12-11

**Authors:** Rebecca M. Koch, Matthijs Kox, Eleonora J. M. Thijs, Janette C. Rahamat-Langendoen, Frank L. van de Veerdonk, Jelle Gerretsen, Joyce Schloesser, Dimitri Diavatopoulos, Guus F. Rimmelzwaan, Mihai G. Netea, Johannes G. van der Hoeven, Marien I. de Jonge, Peter Pickkers

**Affiliations:** ^1^Department of Intensive Care Medicine, Radboud Institute for Molecular Life Sciences, Radboud University Medical Center, Nijmegen, Netherlands; ^2^Radboud Center for Infectious Diseases (RCI), Nijmegen, Netherlands; ^3^Department of Medical Microbiology, Radboud Institute for Molecular Life Sciences, Radboud University Medical Center, Nijmegen, Netherlands; ^4^Department of Internal Medicine, Radboud Institute for Molecular Life Sciences, Radboud University Medical Center, Nijmegen, Netherlands; ^5^NIZO Food Research BV, Ede, Netherlands; ^6^Laboratory of Pediatric Infectious Diseases, Department of Pediatrics, Radboud Institute for Molecular Life Sciences, Radboud University Medical Center, Nijmegen, Netherlands; ^7^Department of Viroscience, Erasmus Medical Center, Rotterdam, Netherlands

**Keywords:** sepsis, lipopolysaccharide, endotoxin tolerance, influenza, live-attenuated quadrivalent influenza vaccine, Fluenz, two-hit model

## Abstract

**Introduction:**

The effects of bacterial infections on the response to subsequent viral infections are largely unknown. This is important to elucidate to increase insight into the pathophysiology of bacterial and viral co-infections, and to assess whether bacterial infections may influence the course of viral infections.

**Methods:**

Healthy male subjects received either bacterial endotoxin [*Escherichia coli-*derived lipopolysaccharide (LPS), 2 ng/kg, *n* = 15] or placebo (*n* = 15) intravenously, followed by intranasal Fluenz (live-attenuated influenza vaccine) 1 week later.

**Results:**

LPS administration resulted in increased plasma cytokine levels and development of endotoxin tolerance *in vivo* and *ex vivo*, illustrated by attenuated cytokine production upon rechallenge with LPS. Following Fluenz administration, infectivity for the Fluenz A/B strains was similar between the LPS–Fluenz and placebo–Fluenz groups (13/15 subjects in both groups). Also, the Fluenz-induced increase in temperature and IL-6, G-CSF and IP-10 concentrations in nasal wash were similar between both groups.

**Conclusion:**

While endotoxemia profoundly attenuates the immune response upon a second LPS challenge, it does not influence the Fluenz-induced immune response. These results suggest immune suppression after bacterial infection does not alter the response to a subsequent viral infection.

## Introduction

Secondary infections with a pathogen other than that which caused the primary infection are generally associated with an unfavorable prognosis compared with *de novo* infections (1). It may be rational to hypothesize that this is the consequence of immunological interplay between pathogen-specific pathways. Concerning the innate immune response, two phenomena have been described for these interactions. First, the primary challenge can induce immunosuppression or “tolerance.” This phenotype is increasingly recognized as the overriding immune dysfunction in bacterial sepsis, where it is known as sepsis-induced immunoparalysis, rendering patients unable to clear their primary infection and rendering them more susceptible toward secondary infections (2). We have previously demonstrated that human endotoxemia [intravenous challenge with bacterial lipopolysaccharide (LPS) in healthy volunteers] results in the development of endotoxin tolerance, exemplified by a severely blunted immune response upon a second LPS challenge (3, 4). Second, the primary challenge can induce “priming” (5) or “training” (6), resulting in a more pronounced response following the secondary infection. As an example for that, bacille Calmette–Guerin vaccination results in enhanced immunological responses by cells of the innate immune system upon a subsequent challenge with a different pathogen (7).

Viral–bacterial interactions are well characterized in animal models, mainly using influenza infection followed by a challenge with live bacteria or LPS. These studies have demonstrated an initial influenza-induced hyperinflammatory state (priming) (8, 9), followed by an immunosuppressive state, which predisposes to secondary bacterial infections (10–12). By contrast, bacterial–viral immunological interactions are much less well studied, and no human data exist to date. The few *in vitro* studies that investigated this interplay have employed co-infection models, in which pretreatment with LPS takes place ≤24 h before influenza infection (13–15). In these studies, LPS pretreatment was shown to induce a primed response upon influenza challenge, characterized by the initiation of an enhanced type I immune response and decreased viral transcription (13–15). Furthermore, it was shown recently that commensal bacteria producing LPS significantly reduce the thermal stability of IAV *in vitro*, that LPS decreases stability of human and avian viral strains at physiological temperatures, and that LPS binds to and affects the morphology of influenza virions (16). In accordance with these *in vitro* data, intramuscular LPS injection in chicken followed by an intranasal influenza challenge 24 h later resulted in reduced influenza viral shedding 4 and 7 days later, compared with animals that did not receive LPS pretreatment. This was accompanied by pulmonary upregulation of interferon (IFN)-α and IFN-γ genes (17). IFNs are known for their antiviral functions, such as inhibition of viral replication and activation of immune cells. However, their upregulation has also been associated with immunosuppression and the increased incidence of secondary infections (18).

In this study, we investigated the bacterial–viral interplay in humans *in vivo* in a unique two-hit model: human endotoxemia followed by a challenge with the mucosal live-attenuated influenza vaccine Fluenz. We used this approach as a model to assess the effects of a bacterial-induced immune response and development of endotoxin tolerance on the response to a subsequent infection with influenza, which is administered in another body compartment: the respiratory mucosa.

## Materials and Methods

### Subjects

This randomized placebo-controlled study was registered at ClinicalTrials.gov (NCT02642237). After approval by the local medical ethics committee (CMO 2015/2058), 30 healthy, non-smoking male subjects aged 18–35 years gave written informed consent to participate in the study. All study procedures were in accordance with the declaration of Helsinki, including the latest revisions. Subjects were screened before the start of the study and had a normal physical examination, electrocardiography, and routine laboratory values. Subjects were excluded if they had a preexistent (lung) disease, a suspicion of influenza infection in the preceding year or a (febrile) illness within 4 weeks before the LPS/placebo challenge. Subjects were not allowed to take (prescription) drugs and to have received a vaccination in the previous months. Subjects were asked to refrain from caffeine and alcohol intake 24 h, and from food 12 h before the LPS/placebo challenge.

To illustrate the development of *in vivo* endotoxin tolerance, we used cytokine data from our previously published double-blind placebo-controlled randomized study (March–April 2012) in which the same inclusion criteria were used, healthy male subjects were administered endotoxin twice with an interval of 1 week [NCT01374711 (3)]. Only data from the six subjects that received placebo beside the endotoxin challenges were used. The human endotoxemia procedures employed in this study are identical to that of our previous study (3).

### Study Design

**Figure 1:**
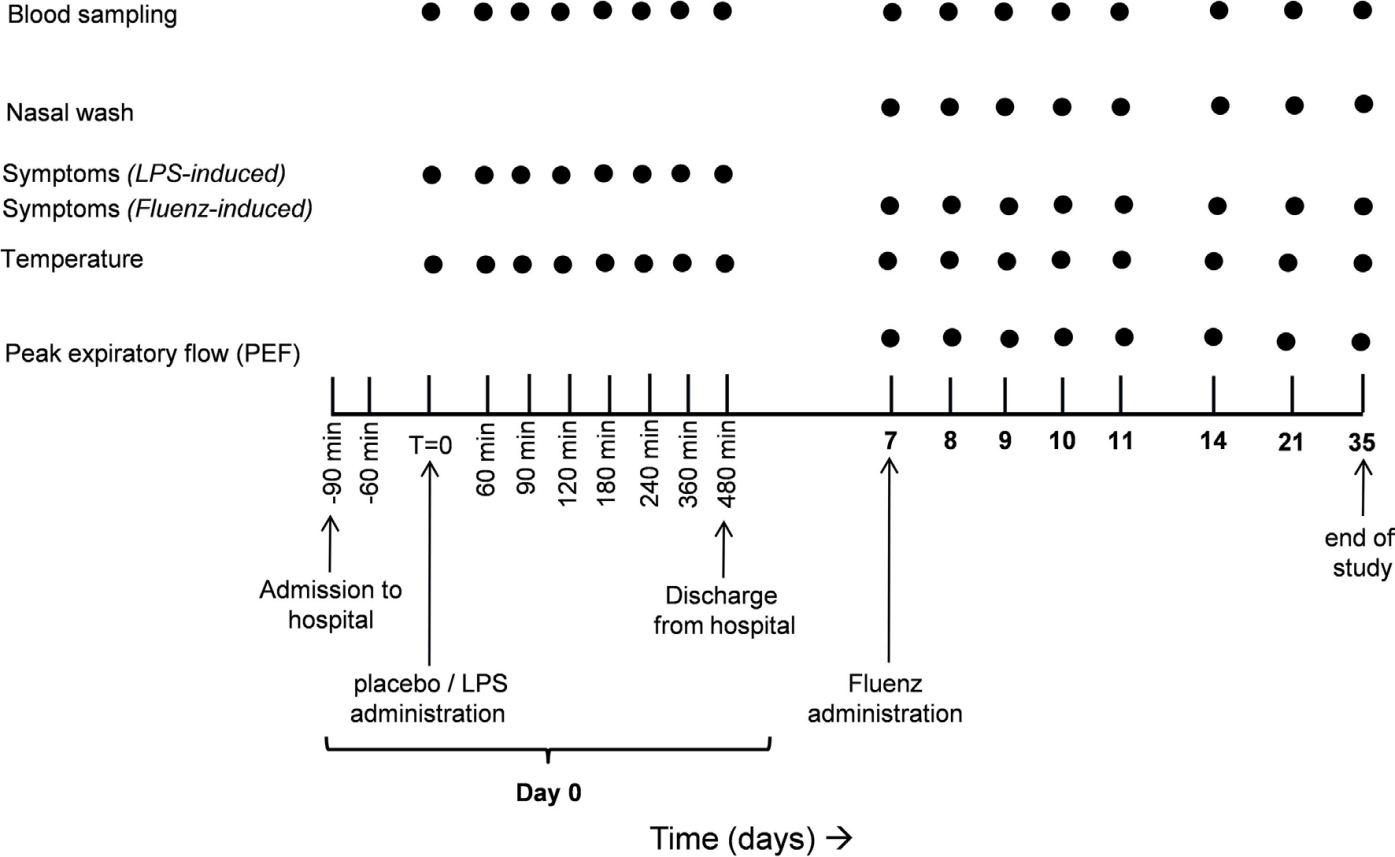
Experimental design.

The study design is depicted in Figure 1. The study was performed from the 11th of January until the 26th of February 2016. Subjects were randomized by an independent nurse to receive an intravenous bolus administration of LPS (2 ng/kg; *n* = 15) or placebo (0.9% saline; *n* = 15) on day 0. The procedures on day 0 were carried out according to our standard human endotoxemia protocol (3). Seven days later (day 7), all 30 subjects underwent intranasal vaccination with the live-attenuated quadrivalent influenza vaccine (LAIV) Fluenz Tetra (0.1 ml/nostril). Subjects remained in the recumbent position for 1 min after Fluenz administration.

Lipopolysaccharide (US Standard Reference Endotoxin *Escherichia coli* O:113), obtained from the Pharmaceutical Development Section of the National Institutes of Health (Bethesda, MD, USA), supplied as a lyophilized powder, was reconstituted in 5 ml saline 0.9% for injection and vortex-mixed for 20 min after reconstitution. Fluenz Tetra (Medimmune LLC, Nijmegen, The Netherlands), complied with the WHO recommendation (Northern Hemisphere) and EU decision for the 2015/2016 season and contained the following strains: A/California/7/2009 (H1N1)pdm09-like, A/Switzerland/9715293/2013 (H3N2)-like strain, B/Brisbane/60/2008 (Victoria lineage)-like strain, B/Phuket/3073/2013 (Yamagata lineage)-like strain, all at 10^7.0±0.5^ FFU*** per 0.2 ml dose.

Fluenz infectivity was defined as positive viral load (measured by real time PCR) in nasal washings for at least one of the influenza strains from day 9 onward and/or seroconversion [defined as ≥4-fold increase in IgG-antibody titer for at least one of the four influenza strains present in the vaccine at 4 weeks postvaccination (day 35) compared with the day of vaccination (day 7)]. For viral load, we assessed Fluenz infectivity from day 9 onward, because Fluenz detected in nasal wash on the first day after vaccination (day 8) most likely originated from the vaccination and does not indicate actual viral replication.

### Nasal Wash

Nasal washings for viral RNA, flow cytometric determinations, leukocyte counts, and cytokine quantification were collected as described previously (19). Nasal wash from two nostrils was pooled and directly analyzed or centrifuged (2,000 *g*, 4°C, 10 min), and stored at −80°C until further analysis.

### Viral Load

**Figure 2:**
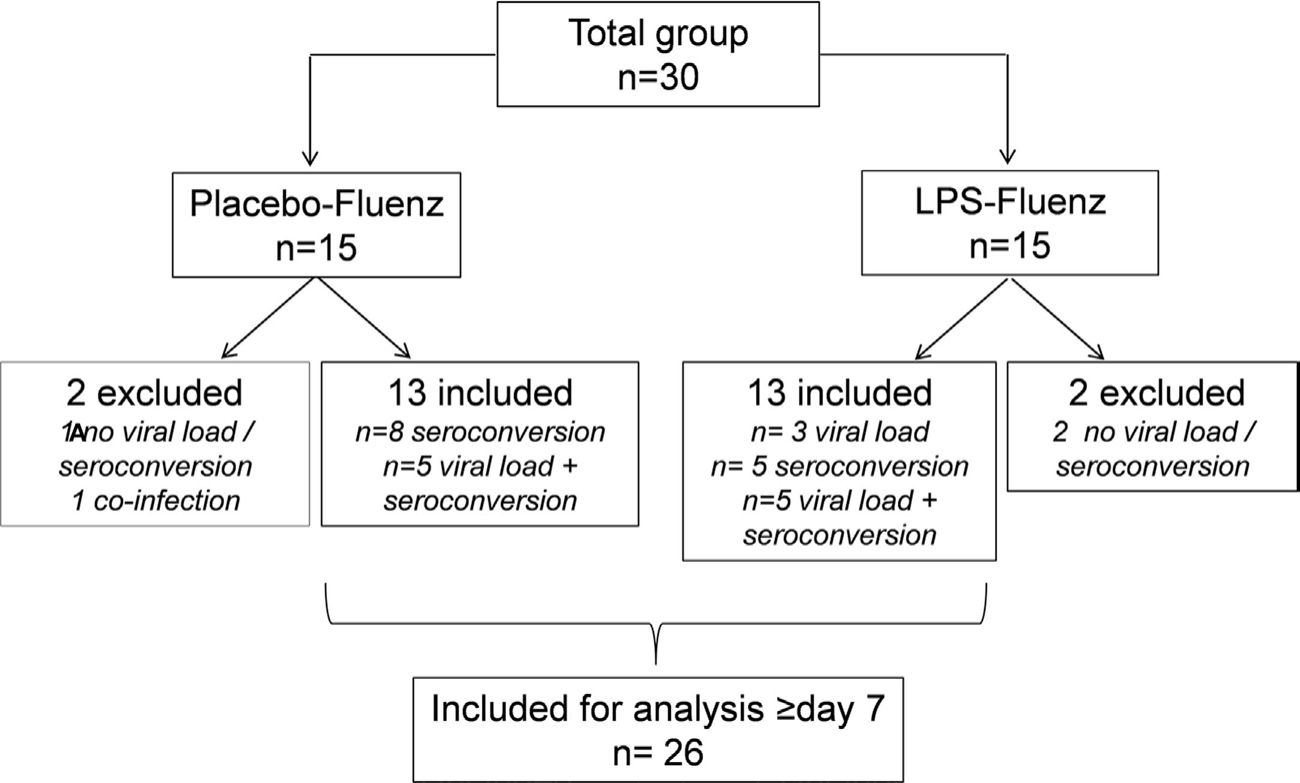
Flowchart based on infectivity, defined as positive viral load for at least one of the influenza strains >day 7 and/or seroconversion (≥4-fold increase in antibody titer for at least one of the influenza strains at day 28 postvaccination compared with baseline). One subject in the placebo–Fluenz group became positive again for one of the influenza strains and was therefore considered to have a co-infection with influenza virus. This subject was excluded for further analyses.

Viral load was semi-quantitatively measured for the Influenza A (with subtype analysis for H1N1 and H3N2) and influenza B strains. Viral load was determined from nasal wash using the MagNA Pure 96 (MagNA Pure 96 DNA and Viral NA Small Volume Kit), and PCRs were performed on the LightCycler 480 with Probes Master Mix (Roche Diagnostics, Almere, The Netherlands) using commercial validated primer and probe mixes (Tib-Molbiol GmbH, Berlin, Germany). Cycling conditions were 95°C for 5 min, followed by 40 cycles of 95°C (15 s), 55°C (15 s), and 72°C (20 s). The relative virus amount was determined based on the difference in cycle threshold value (*C*_t_ value) compared with baseline (day 7), at which virus was undetectable (*C*_t_ > 40, so *C*_t_ was set at 40) in all subjects included for analysis, and expressed as fold change using the formula ${\text{2}}^{\Delta C_{t}}$, where Δ*C*_t_ equals 40 − (*C*_t_ value on day x). The real time PCR for Fluenz was performed at the clinical laboratory of microbiology at the Radboud University Medical Center. This laboratory is ISO certified and participates regularly with QCMD quality controls. The influenza strains measured included: influenza A, influenza B, and influenza A subtypes: influenza A/California/7/2009 (H1N1) and A/Switzerland/9715293/2013 (H3N2). The PCR assay for influenza A and influenza B was more sensitive than the PCR for the influenza A subtypes (H1N1/H3N2), leading to lower *C*_t_ values for influenza A and influenza B. We only measured the influenza A and influenza B strains that were present in the vaccine and no wild-type influenza types, because detection of influenza A and B strains by PCR cannot differentiate between vaccine and wild-type influenza subtypes We excluded subjects in which *C*_t_ values of a particular influenza strain increased again after a period of absence of detectable influenza for this strain, or when the PCR was negative in the first 2 days after vaccination (days 8–9) but became positive in follow-up samples. The latter was the case in one subject (see Figure 2, subject with co-infection). Subjects were also excluded when fever or clinical symptoms appeared for a second time or appeared very late after inoculation with Fluenz. This was also the case in the excluded subject mentioned earlier.

### Serology

Hemagglutination inhibition (HI) assays were performed for the detection of influenza virus specific antibodies. Serum samples were tested for the presence of antibodies to influenza viruses using the HI assay that was performed in duplicate according to standard methods (20, 21). All sera were treated with a filtrate of *Vibrio cholerae* as a source of receptor destroying enzyme and heat inactivated at 56°C. Twofold serial dilutions of the serum samples, starting at 1:20, were incubated with four hemagglutinating units of virus propagated in 11-day-old embryonated chicken eggs for 30 min at 37°C. Subsequently turkey erythrocytes were added, incubated for 1 h at 4°C, and hemagglutination patterns were recorded. For this purpose, vaccine strains NIB-88 [A/Switzerland/9715293/13-like (H3N2)], X-181 [A/California/7/09-like (H1N1)pdm09], B/Phuket/3073/13, or B/Brisbane/060/08 were used. Ferret sera raised against the test antigens were used as positive controls. The paired serum samples of each individual study subject were tested simultaneously. For statistical analysis, a titer of 5 was arbitrarily assigned to sera with a titer <10. Titers were transformed to a logarithmic scale, and geometric means were used for further calculations.

### Hemodynamic Parameters, Symptoms, and Temperature

On day 0, heart rate (3-lead electrocardiogram) and intra-arterial blood pressure data were recorded from a Philips MP50 patient monitor (Philips, Eindhoven, The Netherlands) every 30 s by a custom in-house developed data recording system. On the same day, the LPS-induced rise in temperature and flu-like symptoms (headache, nausea, shivering, muscle pain, and back pain) were scored every 30 min on a 6-point scale (0 = no symptoms, 5 = worst ever experienced), resulting in a total score of 0–25. To assess Fluenz-induced local, lower respiratory tract and systemic symptoms, all subjects filled out an online symptom diary (LimeSurvey Project Hamburg, Germany), using the validated Jackson score [summing the following symptoms: sneezing, nasal discharge, nasal obstruction, sore throat, cough, headache, malaise, and chilliness (22)]. Symptoms were assessed before Fluenz vaccination and then daily until day 28. The severity of each symptom was rated on a 4-point scale. Body temperature was measured using an infrared tympanic thermometer (FirstTemp Genius 2, Sherwood Medical, Crawley/Sussex, UK).

### Peak Expiratory Flow (PEF)

Peak expiratory flow was measured using a peak flow meter PFM20 (Omron Healthcare Europe B.V., Hoofddorp, The Netherlands). PEF was determined twice during each visit and the highest value was used. An affected lower respiratory tract was defined as >20% decrease in PEF compared with the predicted values of subjects’ corresponding age, gender, and stature (23).

### Leukocyte Counts

Analysis of leukocyte counts and differentiation from EDTA anticoagulated blood and nasal wash were measured using routine analysis methods also used for patient samples (flow cytometric analysis on a Sysmex XE-5000).

### *Ex Vivo* Monocyte Stimulation

Primary monocytes were isolated and stimulated as described previously (24). The mononuclear cell fraction was isolated by density centrifugation of EDTA anticoagulated blood, diluted 1:1 in pyrogen-free saline over Ficoll-Paque (GE Healthcare, UK). Isolated cells were washed twice in PBS, and monocyte isolation was subsequently performed using CD14 positive magnetic beads (MACS Miltenyi). MACS isolation was performed according to the manufacturer’s protocol. Monocytes were resuspended in culture medium (RPMI, Invitrogen, Carlsbad, CA, USA) supplemented with 10 µg/ml gentamicin, 10 mM glutamax, and 10 mM pyruvate. Cell counts were performed using a Coulter counter (Coulter Electronics). 1 × 10^5^ monocytes in a 100 µl volume were plated in 96-well flat-bottom plates (Corning, NY, USA) and stimulated for 24 h at 37°C in 5% CO_2_ with 100 µl of RPMI, LPS (10 ng/ml, serotype 055:B5, Sigma-Aldrich), heat-killed *Candida albicans* (1 × 10^6^/ml, strain UC820), and *Staphylococcus aureus* (1 × 10^6^/ml, clinical isolate). Supernatants were stored at −20°C until cytokine analysis.

### Cytokine Analysis

For plasma cytokine concentrations, EDTA anticoagulated blood was centrifuged (2,000 *g*, 4°C, 10 min) and stored at −80°C until analysis. Concentrations of cytokines in plasma, nasal wash, and supernatants of stimulated monocyte cultures were determined by simultaneous Luminex assay (R&D Systems; Abingdon Science Park, UK) and enzyme-linked immunosorbent assays (ELISAs) (R&D Systems, Minneapolis, MN, USA). TNF-α, IL-6, and IL-10 were measured in plasma samples collected on day 0 using a simultaneous Luminex assay (R&D Systems; Abingdon Science Park, UK). In samples obtained from day 7 onward, G-CSF, IL-6, IL-8, IL-10, and IFN-γ in nasal wash were measured using a Luminex assay from R&D Systems (Minneapolis, MN, USA), and IFN-α and IFN-β were measured by a Luminex assay from eBioscience (Vienna, Austria). IP-10 concentrations in nasal wash and plasma were measured using an ELISA (R&D Systems, Minneapolis, MN, USA). Lower detection limits in plasma were 1.2 pg/ml for TNF-α, IL-6, and IL-10, and 156 pg/ml for IP-10. In nasal wash, lower detection limits were 309 pg/ml for IP-10, 0.49 for IFN-α and IFN-β, and 1.4 pg/ml for the remaining analytes. Cytokines in supernatants of *ex vivo* stimulated monocytes were measured using ELISA (IL-1β and IL-13: R&D Systems, Minneapolis, MN, USA, IL-6 and IL-10: Sanquin, Amsterdam, The Netherlands) following the protocols of the manufacturers.

### Statistical Analysis

Data are presented as mean ± SEM, median [IQR] or geometric mean (95% CI). Between-group comparisons were made using Mann–Whitney *U* tests, Kruskal–Wallis tests, Wilcoxon matched-pairs tests, or repeated measures two-way ANOVAs (interaction term) as appropriate, the latter after log transformation if data were not normally distributed (based on the Shapiro–Wilk test). Categorical data were analyzed using Fisher exact tests. Spearman correlation was used. For reasons of clarity, in case of multiple lines in one graph and a logarithmic *y*-axis, only upper or lower bounds of the 95% CI are shown. Statistical analyses were performed using GraphPad Prism version 5.0 (GraphPad Software, San Diego, CA, USA). *p*-Values < 0.05 were considered statistically significant.

## Results

### Demographic Characteristics

Demographic characteristics of the study population are listed in Table 1. We also included the six subjects from our previous study, in which subjects received LPS twice. There were no differences in baseline characteristics between the groups.

**Table 1:** Demographic characteristics of the 30 subjects who participated in the study, including the 6 subjects from our previous study, in which subjects received lipopolysaccharide (LPS) twice.

		LPS–LPS (*n* = 6)		Placebo–Fluenz (*n* = 15)	LPS–Fluenz (*n* = 15)		Total group (*n* = 30)	*p*-Value between groups
Age (years)		21 [20–23]		21 [20–23]	22 [19–23]		21 [20–23]	0.83
Height (cm)		183 [176–189]		180 [178–188]	186 [178–190]		182 [178–189]	0.36
Weight (kg)		81 [75–89]		75 [70–84]	79 [71–87]		78 [69–85]	0.52
BMI (kg/m^2^)		24 [23–26]		23 [20–26]	23 [22–25]		23 [21–25]	0.64

*Parameters were measured during the screening visit*.

*BMI, body mass index*.

*Data are presented as median [IQR]*.

#### LPS/Placebo Challenge

##### LPS-Induced Immune Response *In Vivo*

**Figure 3:**
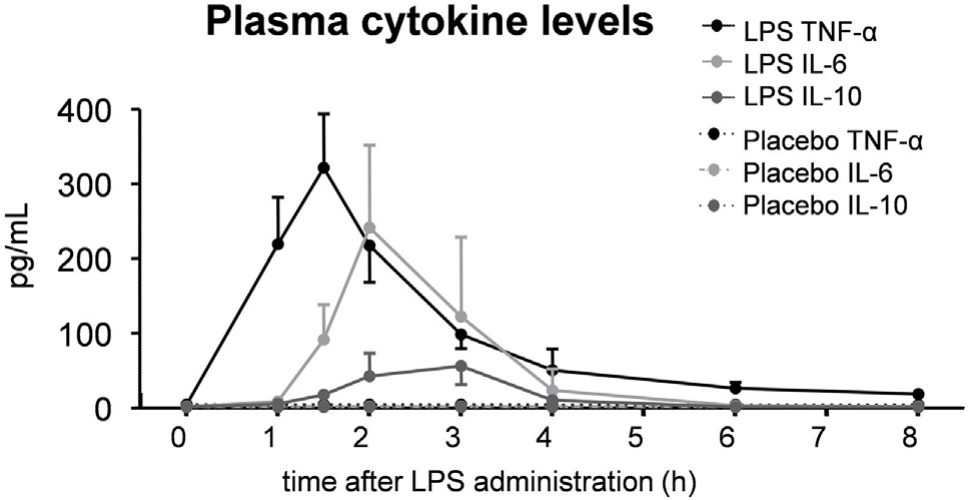
Plasma levels of the cytokines TNF-α, IL-6, and IL-10 in 30 subjects who received an intravenous administration of lipopolysaccharide (LPS) (*n* = 15) or placebo (*n* = 15) at *T* = 0. Data are represented as median [IQR].

**Figure 4:**
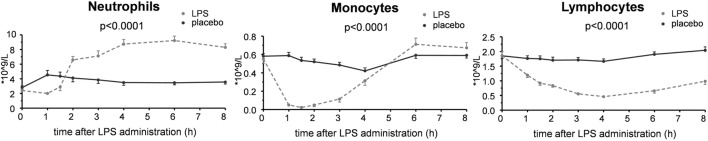
Circulating leukocyte numbers in 30 subjects who received an intravenous administration of lipopolysaccharide (LPS) (*n* = 15) or placebo (*n* = 15) at *T* = 0. Data are represented as mean ± SEM.

**Figure 5:**
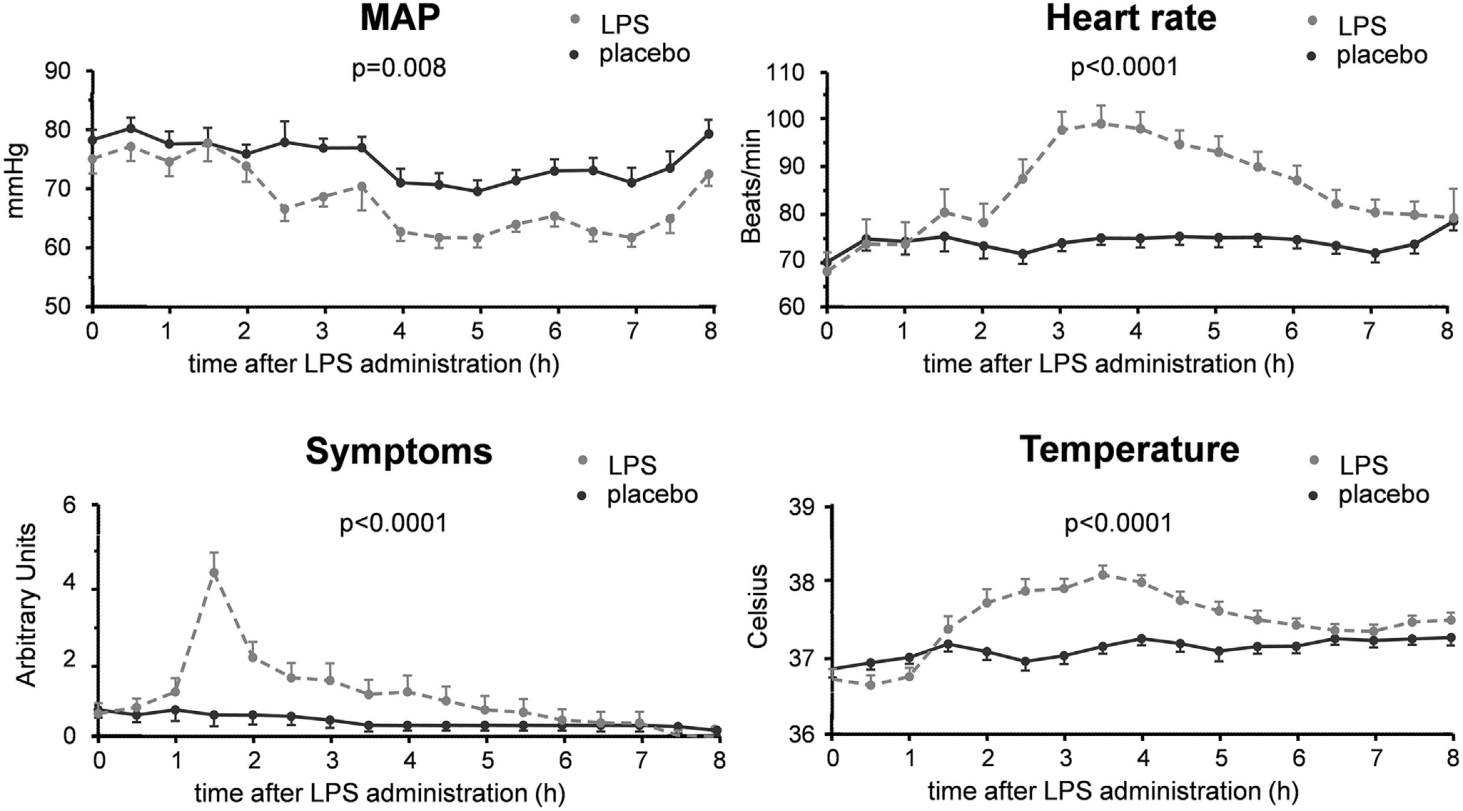
Hemodynamic and clinical parameters in 30 subjects who received an intravenous administration of lipopolysaccharide (LPS) (*n* = 15) or placebo (*n* = 15) at *T* = 0. Data are represented as mean ± SEM.

As expected, plasma levels of TNF-α, IL-6, and IL-10 increased profoundly in the LPS group, but not in the placebo group (Figure 3). This cytokine response was accompanied by a transient monocytopenia, lymphocytopenia, and neutrophilia in the LPS group (Figure 4). In both groups, blood pressure decreased during the experimental day, and this was more pronounced in the LPS than in the placebo group (Figure 5). Only subjects in the LPS group showed an increase in heart rate (Figure 5). LPS-induced symptoms typically started 1 h following LPS administration and peaked at 90 min after LPS administration, accompanied by an increase in body temperature to 38.2 ± 0.1°C (Figure 5).

**Figure 6:**
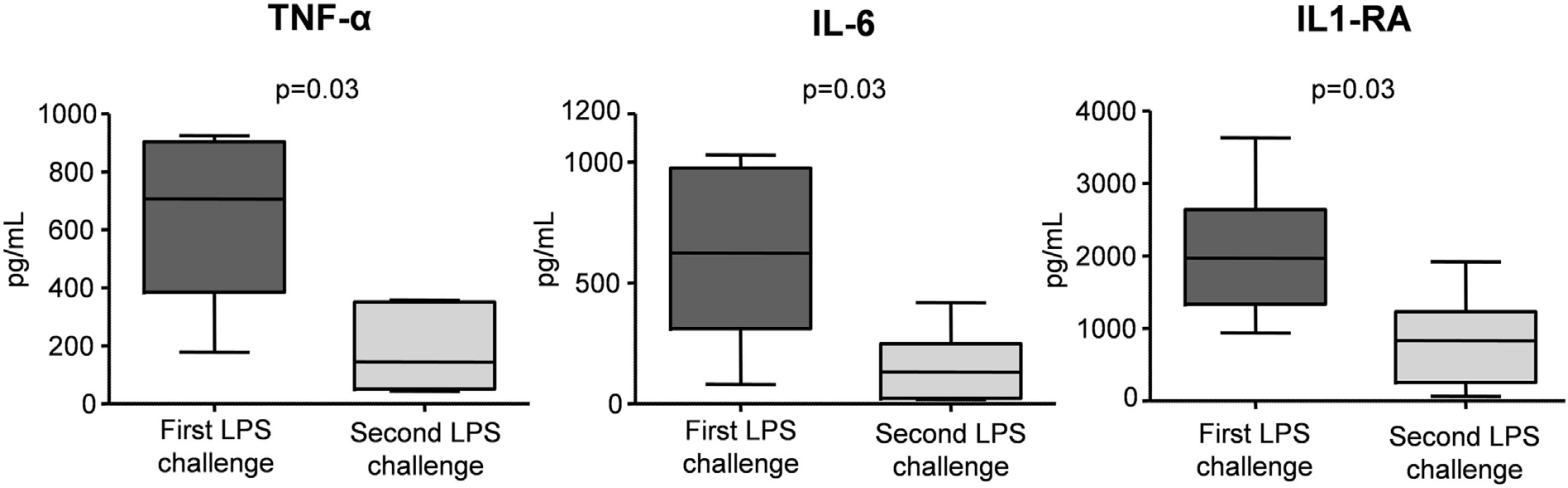
Peak plasma levels of IL-1RA, IL-6, and TNF-α in six subjects who were challenged with lipopolysaccharide (LPS) twice, separated by 1 week. Data are represented as Box and Whisker (Min to Max) plots of individual peak levels of plasma cytokine concentrations during the first and second lipopolysaccharide (LPS) challenge. Data previously published (3).

To illustrate the blunted *in vivo* immune response toward a second LPS challenge 1 week after the first LPS administration, we used data from a previous study in which subjects received LPS twice (with an interval of 1 week) using the exact same endotoxemia protocol as used in this study (3). Upon the second LPS administration, peak plasma levels of the cytokines such as TNF-α, IL-6, and IL-1RA were reduced by a median [IQR] of 74% [61–83], 79% [66–88], and 53% [47–88], respectively, illustrative of profound *in vivo* endotoxin tolerance (Figure 6).

##### *Ex Vivo* Cytokine Responses

**Figure 7:**
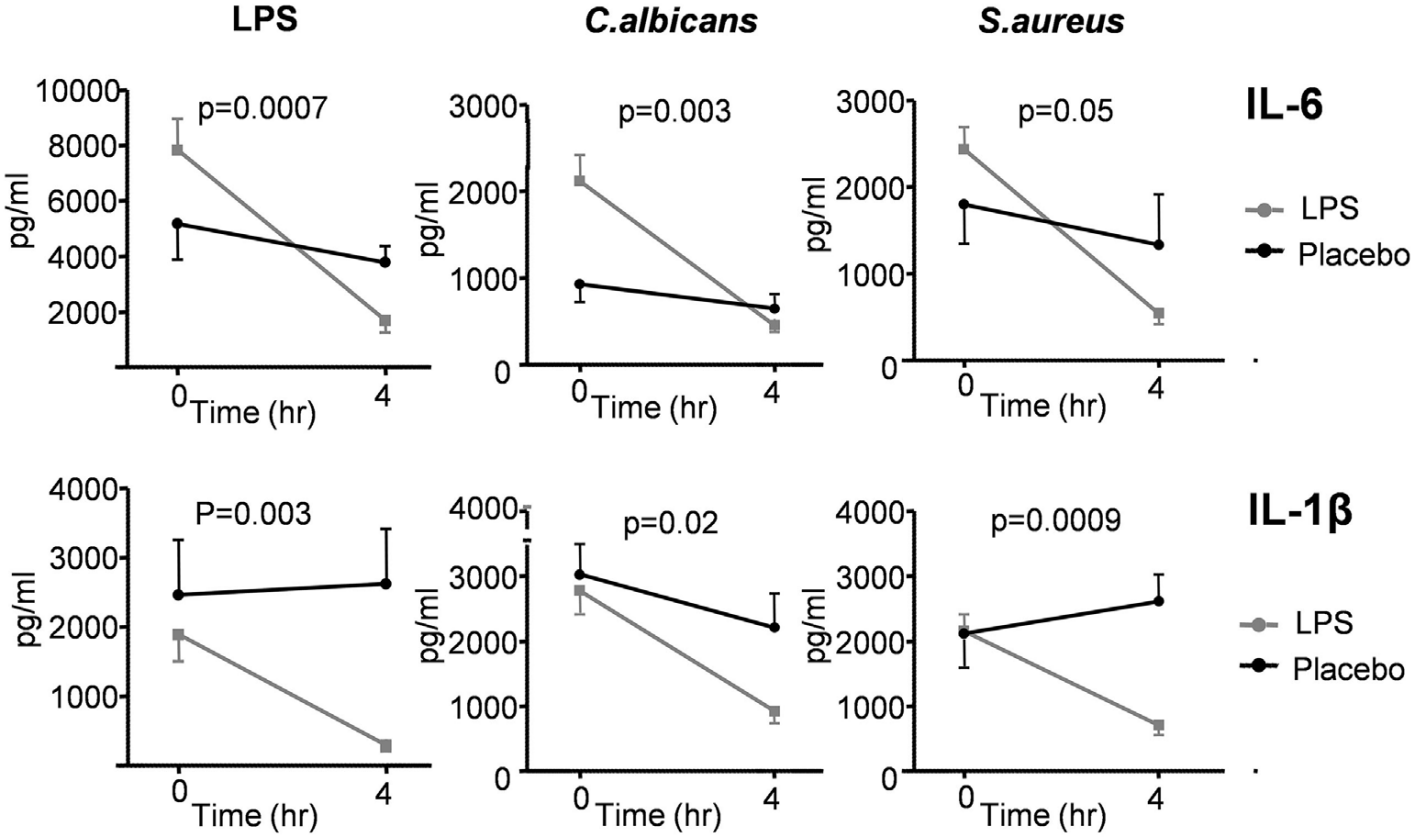
Production of IL-6 and IL-1β measured in supernatants of *ex vivo* stimulated monocytes of 30 subjects who received lipopolysaccharide (LPS) (*n* = 15) or placebo (*n* = 15) at *T* = 0. Monocytes were stimulated with LPS, *Candida albicans*, and *Staphylococcus aureus*. Cytokine production was measured at *T* = 0 (baseline) and 4 h after administration of LPS/placebo. Data are represented as mean ± SEM.

*Ex vivo* stimulation of monocytes with LPS, *C. albicans*, or *S. aureus* demonstrates clear immunosuppression at 4 h after LPS administration, illustrated by significant attenuation of IL-1β and IL-6 production following endotoxemia, compared with the placebo group (Figure 7).

#### Fluenz Challenge

##### Viral Load and Antibody Responses

**Figure 8:**
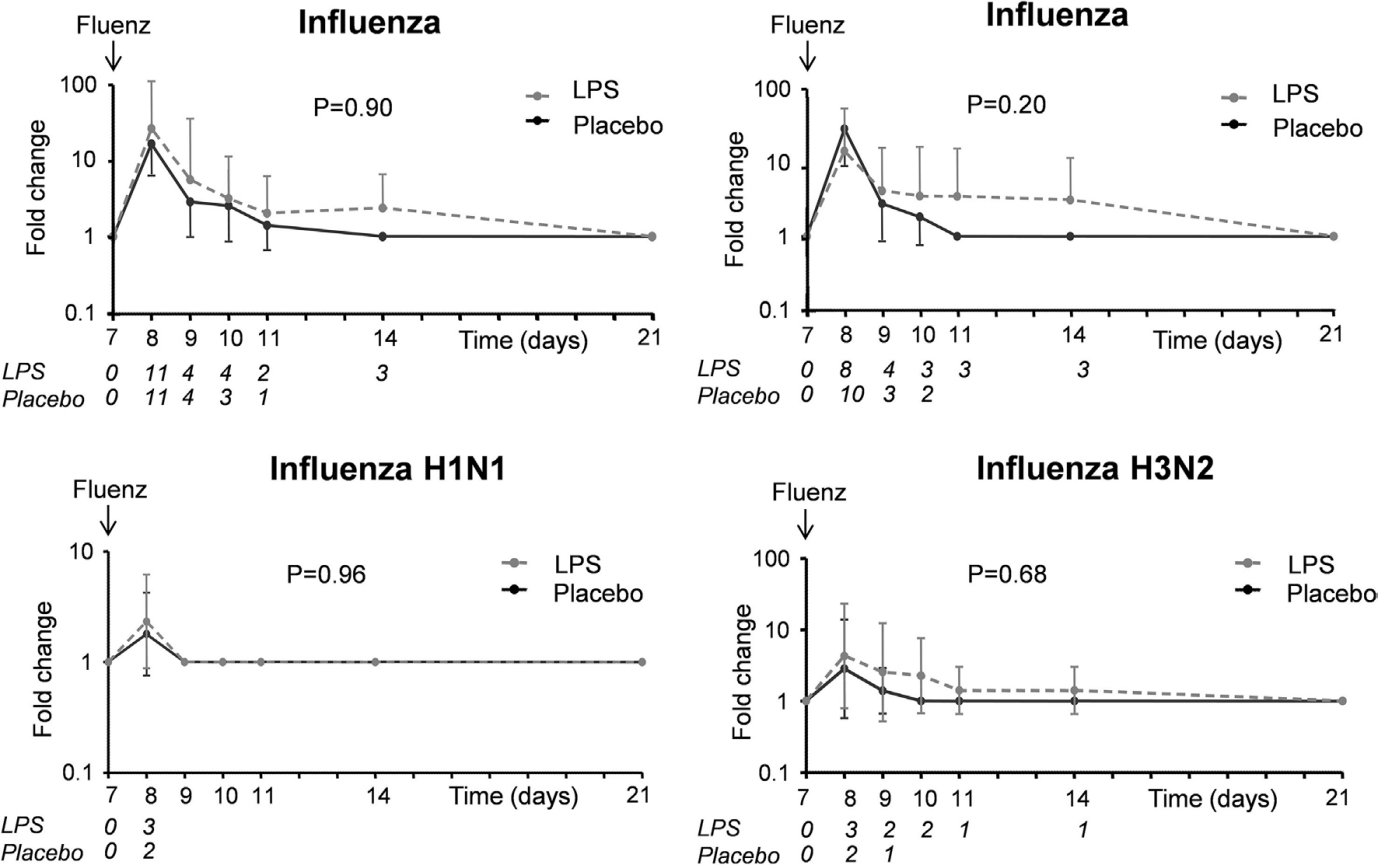
Viral load of the influenza A and B strains as well as the influenza A subtypes H1N1 and H3N2 in nasal wash of the 26 subjects who were challenged with lipopolysaccharide (LPS) (*n* = 13) or placebo (*n* = 13) on day 0 and displayed infectivity after inoculation with Fluenz on day 7. Data are presented as geometric means with 95% CI of fold changes in viral load compared with baseline, where viral load was undetectable (*C*_t_ > 40, so *C*_t_ was set at 40). The italic numbers placed under the time points indicate how many subjects were PCR positive for the respective strain in each group.

**Figure 9:**
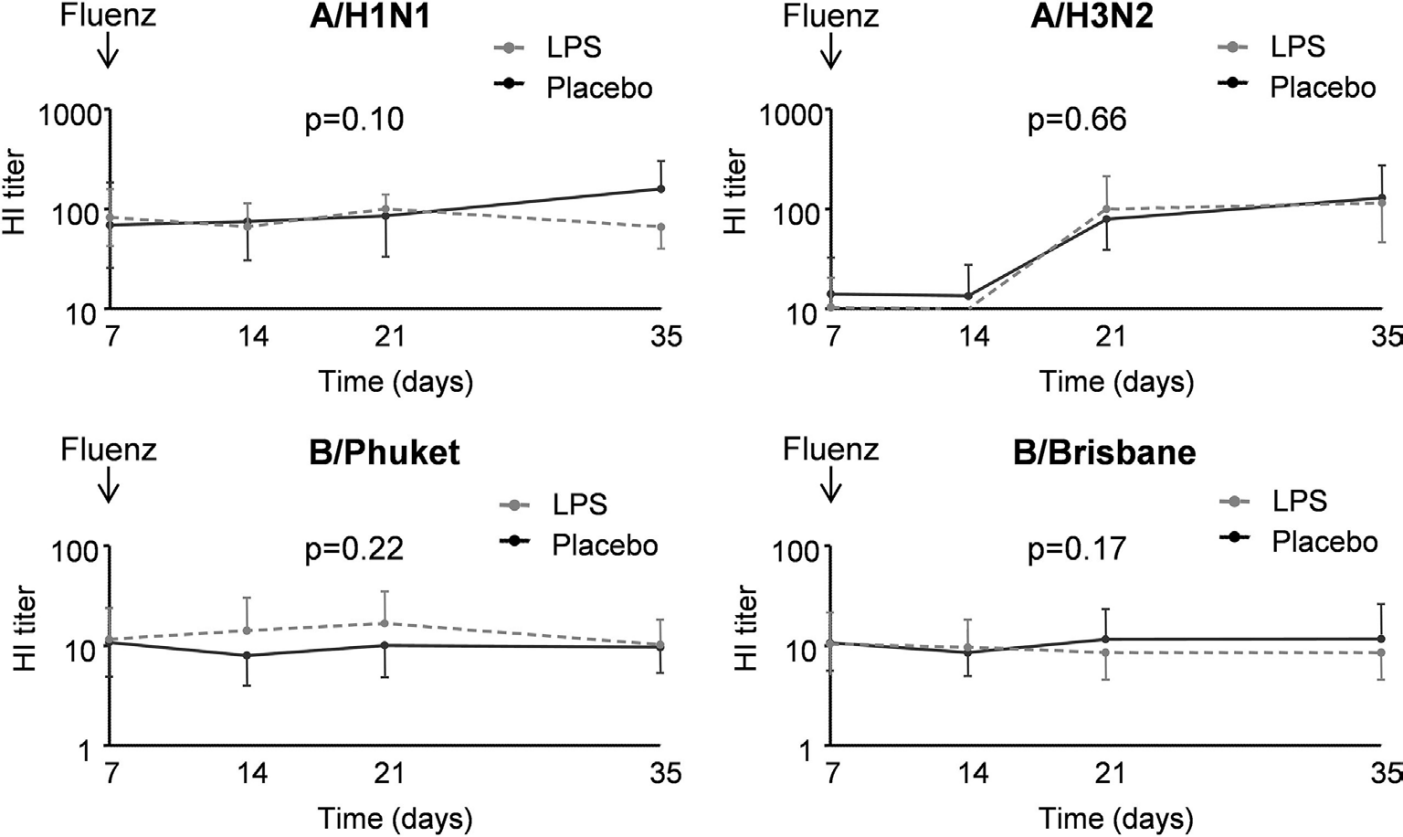
IgG titers for the different influenza strains in 26 subjects who were challenged with lipopolysaccharide (LPS) (*n* = 13) or placebo (*n* = 13) on day 0 and displayed infectivity after inoculation with Fluenz on day 7. Data are represented as geometric mean with 95% CI.

A flowchart of the study based on Fluenz infectivity is depicted in Figure 2. In 26 of the 30 subjects (87%), Fluenz inoculation resulted in infectivity, with identical rates in the LPS–Fluenz and LPS-placebo groups (13 out of 15 subjects in both groups). Three subjects showed no increase in viral load after Fluenz inoculation and one subject in the placebo–Fluenz group showed a second increase in viral load, suggestive for co-infection with a wild-type influenza strain. These subjects were excluded from further analysis (see Figure 2). Changes in viral load are depicted in Figure 8. Influenza A viral load peaked 1 day after Fluenz vaccination to 26 (20–83) and 16 (10–27) fold change in the LPS–Fluenz and placebo–Fluenz group, respectively (*p* = 0.54). Viral load of the influenza B strain peaked to 14 (11–40) and 29 (20–64) fold change in the LPS–Fluenz and placebo–Fluenz group, respectively (*p* = 0.45). Subtyping of the influenza A strain showed only a slight and short-lived increase for the H1N1 strain and a more substantial and sustained increase for the H3N2 strain, with no differences between groups (Figure 8). Viral load gradually returned to baseline levels in the following weeks, with no differences between the LPS and placebo pretreatment groups. Antibody responses over time against the four strains present in the vaccine were also mainly driven by the H3N2 response and comparable between groups (Figure 9). Likewise, the proportion of subjects that displayed seroconversion was similar between groups [10 out of 13 (77%) in the LPS–Fluenz group and all 13 subjects in the placebo–Fluenz group, *p* = 0.22]. Detailed data concerning the antibody responses per strain are listed in Table 2.

**Table 2:** Results of the hemagglutination inhibition (HI) assay for the various influenza strains present in the vaccine in the 26 subjects who were challenged with lipopolysaccharide (LPS) (*n* = 13) or placebo (*n* = 13) on day 0 and were inoculated with Fluenz on day 7.

	Placebo–Fluenz	LPS–Fluenz
Total % seroconversion	13/15 (87%)	10/15 (77%)
**A (H1N1)**		
Baseline HI titer > 1:20 (seropositives)	12/15 (80%)	14/15 (93%)
Seroconversion (day 35)	4/15 (27%)	1/15 (7%)
**A (H3N2)**		
Baseline HI titer > 1:20 (seropositives)	6/15 (40%)	7/15 (47%)
Seroconversion (day 35)	11/15 (73%)	10/15 (67%)
**B (Phuket)**		
Baseline HI titer > 1:20 (seropositives)	6/15 (40%)	7/15 (47%)
Seroconversion (day 35)	0/0 (0%)	0/0 (0%)
**B (Brisbane)**		
Baseline HI titer > 1:20 (seropositives)	6/15 (40%)	5/15 (33%)
Seroconversion (day 35)	2/15 (13%)	0/0 (0%)
GMT H1N1 at baseline	69 (26–184)	82 (43–158)
GMT H1N1 at day 35	158 (83–303)	66 (40–108)
GMT H3N2 at baseline	14 (6–33)	10 (5–21)
GMT H3N2 at day 35	128 (60–275)	115 (46–286)
GMT B (Phuket) at baseline	11 (5–24)	12 (6–24)
GMT B (Phuket) at day 35	10 (5–17)	10 (6–18)
GMT B (Brisbane) at baseline	11 (6–20)	10 (5–22)
GMT B (Brisbane) at day 35	12 (5–26)	9 (5–16)

*GMT, geometric mean titer with 95% CI*.

##### Cytokines and Leukocytes in Nasal Wash

**Figure 10:**
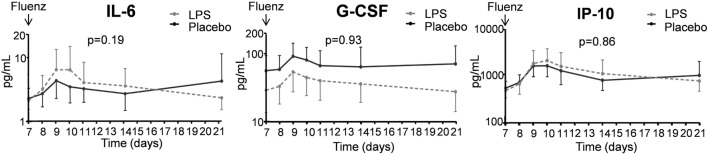
Cytokines in nasal wash in the 26 subjects who were challenged with lipopolysaccharide (LPS) (*n* = 13) or placebo (*n* = 13) on day 0 and displayed infectivity after inoculation with Fluenz on day 7. Data are represented as geometric mean with 95% CI.

**Figure 11:**
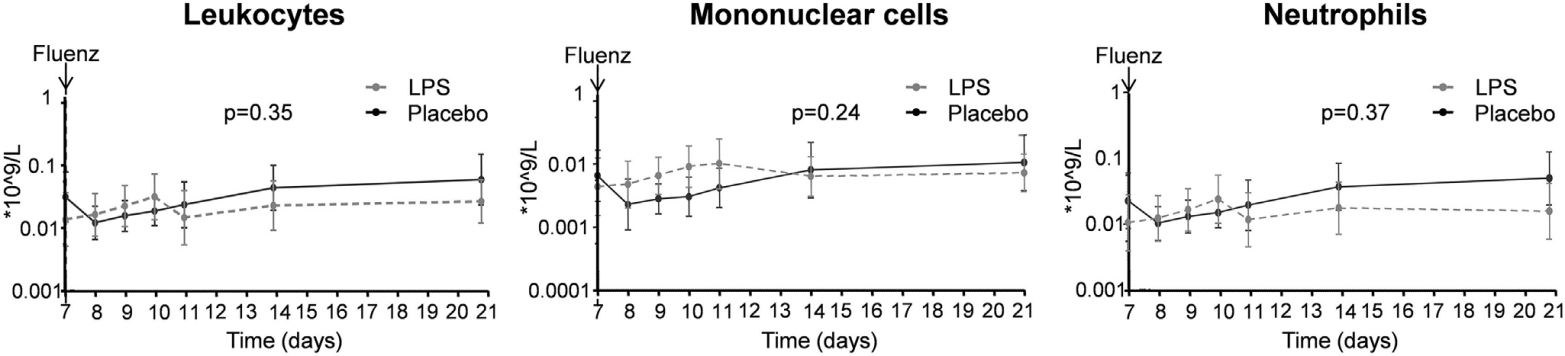
Total leukocyte counts, numbers of mononuclear cells, and neutrophils in nasal wash in the 26 subjects who were challenged with lipopolysaccharide (LPS) (*n* = 13) or placebo (*n* = 13) on day 0 and displayed infectivity after inoculation with Fluenz on day 7. Data are represented as geometric mean with 95% CI.

In both study groups, nasal wash levels of the cytokines IL-6 and G-CSF, and the chemokine IP-10 increased after Fluenz vaccination to a similar extent (Figure 10). The cytokines/chemokines IL-8, IL-10, IFN-α, IFN-β, and IFN-γ were below detection limits in virtually all subjects at all time points, and no clear differences between placebo and LPS groups were observed following Fluenz vaccination. Neither total leukocyte counts nor numbers of mononuclear cells and neutrophils in nasal wash were affected by Fluenz vaccination, and no differences between groups were observed (Figure 11).

##### Local and Systemic Symptoms, Temperature, and PEF

**Figure 12:**
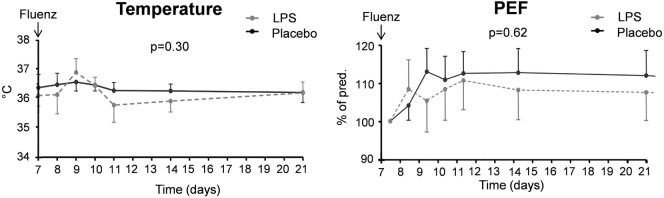
Temperature and peak expiratory flow (PEF) in the 26 subjects who were challenged with lipopolysaccharide (LPS) (*n* = 13) or placebo (*n* = 13) on day 0 and displayed infectivity after inoculation with Fluenz on day 7. Data are represented as geometric mean with 95% CI.

Symptoms indicative for local inflammation of the nasal mucosa in the upper respiratory tract (such as sneezing, nasal discharge, nasal obstruction, sore throat, and cough) did not show a clear increase after Fluenz vaccination and were similar in both groups (maximum levels of 2 [0–5.5] vs. 3 [2–5.5] symptom points in the LPS–Fluenz and placebo–Fluenz groups, respectively, *p* = 0.42). Systemic symptoms (headache, malaise, and chilliness) were not encountered in any of the subjects. Body temperature increased to peak levels of 37.0 [36.9–37.2]°C and 37.2 [36.9–37.3]°C in the LPS–Fluenz and placebo–Fluenz groups, respectively, *p* = 0.52 (Figure 12). Finally, Fluenz vaccination did not affect PEF, as all subjects produced values >80% of their individual predicted values at all time points (Figure 12).

##### Plasma IP-10 Levels and Circulating Leukocytes

Plasma levels of the chemokine IP-10 did not increase following Fluenz vaccination and no differences in circulating leukocyte counts or differentiation were observed (data not shown).

## Discussion

This study demonstrates that a challenge with LPS and the resulting development of profound tolerance to a subsequent challenge with the same agent does not influence the immune response induced by a subsequent viral challenge with the mucosal LAIV Fluenz.

In bacterial sepsis, a tolerant state called “sepsis-induced immunoparalysis” renders the host unable to clear the primary infection and increases the susceptibility toward secondary infections (1). These secondary infections include bacterial and fungal infections, while reactivation of viruses that reside latently in the human host is also frequently encountered (25). However, it was unknown to what extent the development of an endotoxin-tolerant phenotype following bacterial infection influences the innate host response toward a viral challenge. Herein, we employed a unique two-hit model in humans *in vivo*, consisting of a challenge with LPS followed by a Fluenz challenge. This study, as well as previous studies (3, 4, 26–28), show that LPS administration results in the development of endotoxin tolerance *in vivo* and *ex vivo*, illustrated by a profound attenuation of the cytokine response upon a subsequent LPS challenge. As such, human endotoxemia can serve as a model for bacterial sepsis and the associated development of immunoparalysis, and has already been used to investigate potential therapies to reverse sepsis-induced immunoparalysis (3).

The live-attenuated, quadrivalent influenza vaccine “Fluenz Tetra” contains four different influenza strains recommended by international public health agencies as most likely to provide protection against seasonal influenza in any given year (29). Fluenz is a vaccine applied to the nasal mucosa; the natural entrance of respiratory viruses. Therefore, Fluenz vaccination can be used to model influenza infection in humans *in vivo*, albeit a very mild infection, as the response to LAIVs is much less pronounced than to live pathogenic influenza (30). Nevertheless, we believe that LAIVs such as Fluenz are the closest to live pathogenic influenza virus that can be safely used in healthy volunteers.

Our data show that a preceding LPS challenge neither influenced infectivity and innate immune parameters (illustrated by identical cytokine responses in nasal wash) nor impacted measures of adaptive immunity (such as antibody levels and rate of seroconversion), following Fluenz vaccination. These results do not correspond with previous *in vitro* and *in vivo* findings, where immunological priming was observed (13–15, 17). Furthermore, our findings are different than those from to earlier *ex vivo* work, where it was shown that leukocytes from healthy volunteers undergoing human endotoxemia demonstrated a profound tolerant phenotype upon *ex vivo* stimulation with the viral ligands poly(I:C) and S-27609 (28). There are several possible explanations for these seemingly discrepant results.

First, the timing of LPS and influenza challenges may be crucial; in the abovementioned studies, a time interval of 24 h or less between LPS and influenza/viral ligands was employed, whereas we employed an interval of 7 days to ensure that the acute LPS-induced immune response had subsided but endotoxin tolerance was profound. This explanation is supported by two murine studies that showed protection against influenza infection in terms of mortality when LPS was administered 12 and 24 h as well as 3 days before influenza infection, but not when an interval of 7 days was employed (31, 32). These findings in mice strongly suggest that there were no major alterations in the immune response using this interval.

A second possible explanation for the absence of a reduced immune response toward Fluenz after LPS administration is represented by compartment-specific effects. Although *in vivo* endotoxin tolerance is likely due to reprogramming of tissue-resident macrophages, which are assumed to be the main cytokine producers in response to LPS administration *in vivo* (4), it is unknown which tissues are actually affected and to what extent. It can be speculated that the mucosal compartment, in which Fluenz is administered and the initial antiviral immune response mounted, is not tolerized by a preceding LPS administration. To the best of our knowledge, there are no other studies that investigated the effects of a systemic bacterial challenge on a subsequent mucosal challenge. Nevertheless, there are data that show compartment-specific effects concerning endotoxin tolerance. For instance, a study in which murine peritoneal and alveolar macrophages were *ex vivo* restimulated with LPS after a systemic LPS challenge showed a marked discrepancy between these two cell types from different compartments: alveolar macrophages were not displaying endotoxin tolerance, while peritoneal macrophages did (33). The attenuated susceptibility of pulmonary macrophages to develop endotoxin tolerance is supported by other work in mice that were intrapulmonary challenged with 1 µg LPS for four consecutive days, followed by a pulmonary LPS challenge with 10 µg 24 h later. Although TNF-α levels were attenuated in chronic LPS-exposed mice upon the final LPS challenge compared with PBS-pretreated mice, IL-6 levels were increased, accompanied by unrestricted neutrophil recruitment to the alveolar space (34). It was speculated that this represents a mechanism by which the lungs protect themselves against pulmonary bacterial infections (34). In addition, chronic pulmonary LPS exposure did not confer cross-tolerance to the TLR2 ligand Pam3Cys (34). Taken together, similar to the lung, the nasal mucosa might be less sensitive to tolerance. This compartmentalization could also explain why primary virus infections entering the body through the nasal mucosa have not been reported following sepsis-induced immunosuppression, while latent viruses that are already present in the systemic compartment may induce infection in the immunocompromised host through reactivation.

Third, one may argue that the absence of an effect of preceding LPS administration on antiviral responses in our study might be that the human endotoxemia model and the resulting immunosuppressive effect is too mild to affect antiviral immunity. However, the profound (>70%) suppression of the response to a subsequent LPS challenge, also 1 week after the first LPS challenge, indicates that this model is able to induce clinically relevant tolerance in case of rechallenge with LPS.

Finally, the pathophysiology induced by the use of the LAIV Fluenz may not be comparable with the actual pathogenic influenza virus, because it is an attenuated virus that may not show same infectivity, tissue tropism and virus dissemination as actual influenza. Furthermore, the immune evasion strategies/other specific immune parameters of live virus in the host cells might not be shown by the LAIV, as discussed elsewhere (35, 36). Nevertheless, aforementioned work in mice using a pathogenic influenza strain (31, 32) corroborates our findings that an interval of 7 days does not result in an altered response upon challenge with influenza. As alluded before, we believe that using LAIVs such as Fluenz is the most accurate way to model an actual influenza infection in humans *in vivo* in a safe manner. Use of a primate model (37–39) could definitively exclude that the lack of effects by LPS pretreatment is not due to differences between LAIVs and pathogenic influenza virus.

The lack of an *in vivo* interaction found in this study might explain why no adverse effects of the live-attenuated influenza vaccine have been reported in immunocompromised patients, including the elderly and young children (40–43), while immunogenicity of the vaccine is unaltered (40, 43). This is demonstrated by the absence of an exaggerated immune response or excessive viral replication, and seroconversion rates similar to those observed in healthy young adults. Nevertheless, the vaccine is not recommended for immunocompromised patients (29).

The Fluenz-induced immunological effects observed in this study are in accordance with previous work (29, 44–48). The proportion of subjects that displayed detectable influenza virus in nasal washings after Fluenz vaccination (47), as well as the increased production of cytokines in nasal wash (45) is in line with previous findings. Moreover, Fluenz-induced robust serum antibody responses (46), especially for the H3N2 strain (29). The overall high seroconversion rate in our study could be explained by our study population, which are young, healthy males with competent immune systems, efficient in eliminating Fluenz.

A limitation to our study is the fact that we performed the study during the winter season could have influenced the results. Although we tested for the presence of various influenza strains, other respiratory viruses are prevalent in the winter period as well, such as the human rhinovirus and respiratory syncytial virus, and it has been demonstrated that viral co-infections may alter the disease course (49–51). Also, the reactivation of latent viruses, such as herpes simplex virus, CMV, and Epstein–Barr virus are common in this season, which could have affected the immune response as well (52, 53).

In conclusion, challenge with the bacterial ligand LPS does not affect the mediated response toward a subsequent viral challenge consisting of the live-attenuated influenza vaccine Fluenz. Our results suggest that immune suppression after bacterial infection does not alter the response to a subsequent viral infection.

## Ethics Statement

This randomized placebo-controlled study was registered at ClinicalTrials.gov (NCT02642237). After approval by the local medical ethics committee (CMO 2015/2058), 30 healthy, non-smoking male subjects aged 18–35 years gave written informed consent to participate in the study. All study procedures were in accordance with the declaration of Helsinki, including the latest revisions.

## Author Contributions

RK designed and performed the study, analyzed and interpreted data, and wrote the manuscript. MK supervised and designed the study, interpreted data, and edited the manuscript. JS, DD, FV, GR, JR-L, and ET performed measurements and interpreted data. JG performed laboratory measurements. JH was chief of department and supervised the project. MN and MJ interpreted data and reviewed the report. PP was the principal investigator, designed the study, interpreted data, and reviewed the report.

## Conflict of Interest Statement

The authors declare that the research was conducted in the absence of any commercial or financial relationships that could be construed as a potential conflict of interest.
